# Association of Sedentary Behavior Time with Ideal Cardiovascular Health: The ORISCAV-LUX Study

**DOI:** 10.1371/journal.pone.0099829

**Published:** 2014-06-12

**Authors:** Georgina E. Crichton, Ala'a Alkerwi

**Affiliations:** 1 Nutritional Physiology Research Centre, University of South Australia, Adelaide, Australia; 2 Centre de Recherche Public Santé, Centre d'Etudes en Santé, Grand-Duchy of Luxembourg; Bielefeld Evangelical Hospital, Germany

## Abstract

**Background:**

Recently attention has been drawn to the health impacts of time spent engaging in sedentary behaviors. No studies have examined sedentary behaviors in relation to the newly defined construct of *ideal cardiovascular health*, which incorporates three health factors (blood pressure, total cholesterol, fasting plasma glucose) and four behaviors (physical activity, smoking, body mass index, diet). The purpose of this study was to examine associations between sedentary behaviors, including sitting time, and time spent viewing television and in front of a computer, with cardiovascular health, in a representative sample of adults from Luxembourg.

**Methods:**

A cross-sectional analysis of 1262 participants in the Observation of Cardiovascular Risk Factors in Luxembourg study was conducted, who underwent objective cardiovascular health assessments and completed the International Physical Activity Questionnaire. A Cardiovascular Health Score was calculated based on the number of health factors and behaviors at ideal levels. Sitting time on a weekday, television time, and computer time (both on a workday and a day off), were related to the Cardiovascular Health Score.

**Results:**

Higher weekday sitting time was significantly associated with a poorer Cardiovascular Health Score (*p* = 0.002 for linear trend), after full adjustment for age, gender, education, income and occupation. Television time was inversely associated with the Cardiovascular Health Score, on both a workday and a day off (*p* = 0.002 for both). A similar inverse relationship was observed between the Cardiovascular Health Score and computer time, only on a day off (*p* = 0.04).

**Conclusion:**

Higher time spent sitting, viewing television, and using a computer during a day off may be unfavorably associated with ideal cardiovascular health.

## Introduction

Cardiovascular disease (CVD) remains the leading causes of mortality in the United States and Europe [1], [2]. Preventing or lowering adverse levels of risk factors may be the most effective means for averting clinical events in at-risk individuals, and reducing overall CVD prevalence. The American Heart Association (AHA) has recently established a construct of ‘ideal cardiovascular health’ [3]. The simultaneous presence of four positive health behaviors (non-smoking, achieving recommended levels of physical activity, maintaining normal body mass index [BMI], and healthy diet), and three health factors (total cholesterol, blood pressure, and fasting blood glucose at goal levels) has been shown to be a strong predictor of mortality from all causes and from CVD [4], [5].

While physical activity, one component of the ideal cardiovascular health construct, has been inversely related to fatal and nonfatal CVD [6]–[11], attention has more recently been drawn to the association between sedentary behaviors and cardiovascular risk factors and CVD [12]–[16]. Sedentary behavior refers to any waking behavior that involves an energy expenditure of less than 1.5 metabolic equivalent units (METs) [17], [18]. This can include activities undertaken in sitting or lying down, such as watching television or using a computer. While individual studies have shown relationships between sedentary behaviors and negative health outcomes [19]–[25], there are some inconsistencies in the literature [26], [27] and several reviews have concluded that causal relationships between sedentary behavior time and health outcomes need to be further clarified [14], [15].

Only few studies to date have looked at associations between time spent in sedentary behaviors and a cluster indicator of risk for CVD [21] or metabolic syndrome [28]. One such cluster indicator is the metabolic syndrome, which has shown to be a good predictor for cardiovascular health outcomes [28]–[32]. However, the practical utility of metabolic syndrome as a diagnostic or management tool and use in public health research has been questioned [33], [34]. Of particular note is that the metabolic syndrome, like other CVD risk prediction scales, such as the Framingham Cardiovascular Disease Risk Profile [35], does not include health behaviors such as diet or physical activity [36]. A cluster indicator for cardiovascular health gives the opportunity to research the joined effect of interrelated risk factors, by focusing on an overall state of health, rather than disease or mortality risk.

Most of the literature to date on sedentary behavior and health outcomes, most frequently, mortality and CVD risk, have concentrated on television viewing time. However, other sedentary behaviors may or may not have a similar impact upon health outcomes [37], such as time during transportation, time sitting at work either at a computer or not, sitting at meal times, or engaging in other leisure activities such as reading. Few studies have considered the independent associations of different sedentary behaviors with cardiovascular outcomes, and none that we are aware of, with a global indicator of cardiovascular health, comprising both health factors and lifestyle behaviors, in a general healthy population sample.

The first aim of this study was to investigate weekday sitting time, including various sedentary behaviors, in relation to a global indicator of cardiovascular health [3], comprising seven health metrics: BMI, smoking, diet, physical activity, fasting plasma glucose, total cholesterol, and blood pressure. The second aim was to explore relations between both television time and computer time with levels of ideal cardiovascular health, and whether there were any differences according to day of the week; i.e. between a workday and a day off. It was hypothesised that there would be inverse associations between all measures of sedentary behaviors (sitting time, television time, computer time) and ideal cardiovascular health. We did not advance a hypothesis as to how any relationships may vary according to day of the week.

## Methods

### Ethical Statement

All participants gave informed written consent to take part in the study. The study design and information collected were approved by the National Research Ethics Committee (Comité d'Ethique de Recherche, CNER) and the National Commission for Private Data Protection (Commission Nationale pour la Protection des Données, CNPD).

### Participants

Data was obtained from the cross-sectional Observation of Cardiovascular Risk Factors in Luxembourg (ORISCAV-LUX) study, conducted between November 2007 and January 2009. ORISCAV-LUX was designed as a nationwide cardiovascular monitoring survey to establish information on the prevalence of cardiovascular risk factors, including obesity, hypertension, diabetes mellitus, and dyslipidemia, among the general adult population of Luxembourg. A representative random sample of 4496 individuals, stratified by sex, age (18–69 years) and district of residence, was selected from the national health insurance registry, to ensure statistical power, i.e. a statistical precision of at least 2% for the estimation of the prevalence of the risk factors at the 95% confidence level. The only exclusion criteria were those who were institutionalized (*n* = 12), pregnant (*n* = 21), with serious mental and/or physical handicap (*n* = 5), prisoners (*n* = 1), people outside the determined age range (*n* = 2) and those deceased before recruitment (*n* = 5). Description of the recruitment and sampling scheme have been published in detail previously [38], [39]. A total of 1432 participants completed the recruitment procedure, with a response rate of 32.2%, which corresponded to the expected rate upon which the sample size was calculated. After eliminating those with missing data on components of cardiovascular health, sedentary time or covariates, data from 1266 participants were available for analysis. A further four participants were excluded who reported implausible television or computer times (>18 hours on a workday or day off). The final sample consisted of 1262 participants.

### Procedure and Measures

#### Demographics and health information

A detailed self-administered questionnaire was used to gain information on demographic and socioeconomic characteristics, including age, gender, education, occupation, and income. Education level was classified into three levels, based on the highest diploma obtained: ‘primary’ (less than 12 years of education), ‘secondary’ (approximately 12 to 13 years of education) and ‘tertiary’ (more than 13 years). Participants were required to indicate their type of occupation from 1 of 14 areas. As we are interested in the association between time spent sedentary and health outcomes, the 14 areas of occupation were categorized into three main groups: ‘sedentary’, ‘moderately active’, and ‘active’, estimated based on the type of work performed. For example, manual laborers were placed in the active category, while scientific professionals were placed in the sedentary category. Economic status was ascertained by asking participants to select the category best representing total monthly household income and to indicate the number of adults and children living in the same household, in order to measure the Adult Equivalent Income (AEI). On the basis of the current official national poverty risk threshold for AEI (National Institute of Statistics), the income variable was classified as either above or below the poverty risk threshold.

#### Cardiovascular health assessment

Detailed data regarding cigarette smoking were obtained from the questionnaire. Each participant was classified as current smoker, ex-smoker or non-smoker. Dietary intake was assessed using a semi-quantified food frequency quesionnaire (FFQ) which assesses the frequency of consumption and portion size of 134 items over the previous three months. Specifically, intakes of fruits and vegetables, fish, fiber-rich whole grains, sodium and sugar-sweetened beverages were extracted from the questionnaire in order to calulate a ‘healthy diet score’ as defined by the AHA [3]. The diet score, ranging from 0 to 5, is dependent upon meeting recommended intakes of the forementioned foods and beverages, consistent with the current Dietary Guidelines of Americans [40]. Physical activity was assessed using the short-form International Physical Activity Questionnaire (IPAQ) [41], designed to measure physical activity in large populations. Self-reported time spent engaging in both moderate and intense physical activity was used to calculate total physical activity time in minutes per week.

Participants underwent a venous blood sample draw following an overnight 8-hour fast. Blood samples were transferred to the laboratory of the ‘Centre Hospitalier in Luxembourg’ for analysis. Laboratory tests performed included fasting plasma glucose (FPG, mg/dl), triglycerides (TG, mg/dl), total cholesterol (TC, mg/dl), low-density lipoprotein cholesterol (LDL-C, mg/dl), high-density lipoprotein cholesterol (HDL-C, mg/dl), and C-reactive protein (CRP, mg/l).

Body weight (kg) was measured using a digital column scale, with subject barefoot and wearing light clothing. Standing body height (cm) was recorded to the nearest 0.2 cm with a portable wall stadiometer attached to the scale, with heels together. BMI was calculated as body weight (kg) divided by height squared (m^2^).

Systolic blood pressure (SBP, mmHg) and diastolic blood pressure (DBP, mmHg) were measured three times in sitting with a minimum 5 minute interval between each measurement, using an automated oscillometric blood pressure monitor. The average of the last two readings was used in the analysis.

#### Cardiovascular Health Score (CHS)

The AHA definitions [3] were used to determine the level of cardiovascular health for the seven individual health metrics. Ideal levels for each metric are as follows: smoking: never or quit >12 months ago, BMI: <25 kg/m^2^, diet score: ≥4 out of 5 recommended dietary items, physical activity: ≥150 minutes per week of moderate intensity activity or ≥75 minutes per week of vigorous intensity activity (or a combination), total cholesterol: <200 mg/dL, BP: <120/<80 mm Hg, and fasting plasma glucose: <100 mg/dL. For each component, participants were given a score of 1 if they met the ideal AHA criterion, otherwise 0 points were assigned. A total CHS was calculated ranging from 0 (no cardiovascular health metric at ideal levels) to 7 (all cardiovascular health metrics at ideal levels).

#### Sedentary behavior time

Time spent sitting, viewing television and using a computer were obtained from the self-report IPAQ [41]. These activities can be regarded as measures of sedentary behaviors, characterized by an energy expenditure of less than 1.5 METs [17]. Participants reported how much time they spent sitting during a normal weekday (distinguished form a weekend day), including time spent sitting at place of work, on transportation, reading, visiting friends', sitting or laying down to watch television or use a computer. They were also asked to report how much time they spent watching television (including videos/DVD), and in front of a computer (including internet and video games), during the course of a typical workday, and during a day off. These questions did not stipulate that the participant had to be sitting while watching television or using a computer, however, the IPAQ is conceived in a way to distinguish the time spent in performing four types of physical behaviors (vigrous physical activity, moderate physical activity, walking and sitting). All responses were given in hours per day, with reference to the preceding seven days.

The following five sedentary time indicators were used as the independent predictor variables: sitting time weekday, television time workday, television time day off, computer time workday, and computer time day off. The ‘sitting time weekday’ variable (in hours per day) was divided into five categories: 0–2, >2–4, >4–6, >6–10, and >10 hours per day. Similarly, the television time and computer time variables, in hours per day, were divided into five categories (0, >0–1, >1–2, >2–3, and >3 hours per day).

### Statistical Analyses

For the descriptive analyses, Chi-squared tests were performed to compare the demographic characteristics of participants according to self-reported weekday sitting time.

General linear modelling with polynomial trend analyses was used to compare the CHS (as a continuous variable, ranging from 0–7) across increasing categories of time for each of the sedentary behavior variables: weekday sitting time, television time workday, television time day off, computer time workday, and computer time day off. Covariates included in all models were age, education, gender, occupation and income.

ANOVA and general linear modelling with polynomial trend analyses were used to compare each indiviudal cardiovascular health metric (and the total CHS) across increasing categories of weekday sitting time. Two models are presented: 1) unadjusted, and 2) adjusted means for age, education, gender, occupation and income.

Several sensitivity analyses were performed to test the robustness of our findings. Firstly, general linear modelling as described above were performed excluding physical activity from the CHS (ranging from 0–6), and including it as covariate. Secondly, linear regression analyses examining the association between CHS and each of the sedentary behaviors as continuous variables (hours per day) was performed.

All statistical anlyses were performed with PASW for Windows® version 21.0 software (formerly SPSS Statistics Inc. Chicago, Illinois). *p*<0.05 was considered statistically significant.

## Results

### Sample Characteristics

The sample consisted of 1262 individuals (619 males and 643 females), aged 18 to 69 years (mean 44±13 years). Mean weekday sitting time was 6.2 (±3.2) hours. Reported television time on a weekday was 1.7 (±1.5) hours, and this increased to 2.4 (±1.8) hours on a day off. Computer time on a workday was 2.5 (±3.1) hours, which decreased to 1.0 (±1.5) hours on a day off.

Table 1 shows the demographic and socioeconomic variables for the ORISCAV-LUX participants according to weekday sitting time (time spent at the office, on transportation, visiting friends', reading, sitting or laying down to watch television or use a computer). Higher weekday sitting time was higher in males, in those with a tertiary education and in more affluent subjects.

**Table 1:** Demographic and socioeconomic characteristics according to weekday sitting time^a^, ORISCAV-LUX (*N* = 1262).

	Weekday sitting time^a^, hours per day										
Characteristic	0–2		>2–4		>4–6		>6–10		>10		*p* b
	*N*	%	*n*	%	*n*	%	*n*	%	*n*	%	
	122	9.7	312	24.7	291	23.1	413	32.7	124	9.8	
**Gender**											<0.001
Males	42	34.4	127	40.7	143	49.1	230	55.7	77	62.1	
Females	80	65.6	185	59.3	148	50.9	183	44.3	47	37.9	
**Education**											<0.001
Primary	46	37.7	98	31.4	93	32.0	67	16.2	20	16.1	
Secondary	60	49.2	164	52.6	136	46.7	179	43.3	53	42.7	
Tertiary	16	13.1	50	16.0	62	21.3	167	40.4	51	41.1	
**Occupation**											<0.001
Sedentary	4	3.3	14	4.5	12	4.1	23	5.6	6	4.8	
Moderately active	74	60.7	216	69.2	207	71.1	338	81.8	105	84.7	
Active	44	36.1	82	26.3	72	24.7	52	12.6	13	10.5	
**Income**											<0.001
Below poverty threshold risk	42	40.4	82	29.2	49	19.8	43	11.8	16	14.0	
Above poverty threshold risk	62	59.6	199	70.8	199	80.2	320	88.2	95	86.0	

a Includes time spent sitting at place of work, on transportation, at friends', reading, sitting or laying downto watch television or use a computer.

b Chi-square for categorical variables.

### Weekday Sitting Time and Cardiovascular Health

**Figure 1:**
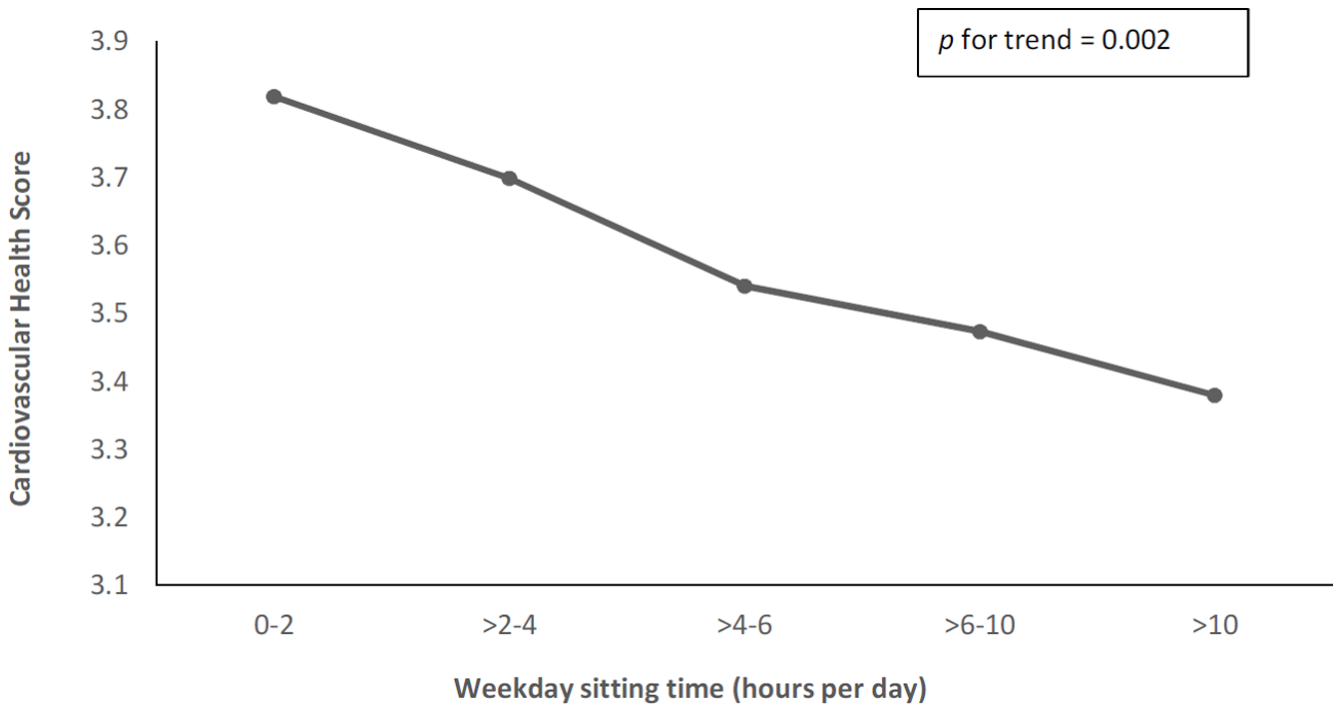
Multivariate adjusted means for Cardiovascular Health Score according to weekday sitting time. Sitting time includes time spent during transportation, at place of work, watching television, and in front of a computer. Means are adjusted for age, gender, education, income, and occupation.

The CHS decreased significantly as weekday sitting time increased (Table 2 and Figure 1; *p* = 0.002 for linear trend), after full adjustment for age, gender, education, occupation and income. For the individual metrics, physical activity was significantly negatively associated with sitting time (*p*<0.001, adjusted model). Similarly, healthy diet scores decreased (reflecting poorer diet), as weekday sitting time increased (*p* = 0.001, adjusted model). Positive associations were observed between SBP (*p* = 0.017, unadjusted model) and total cholesterol (*p* = 0.05, adjusted model) and the CHS.

**Table 2:** Cardiovascular health metrics according to weekday sitting time^a^, ORISCAV-LUX (*N* = 1262).

Health metric	Model^b^	Weekday sitting time^a^, hours per day										
		0–2		>2–4		>4–6		>6–10		>10		*P* c
		Mean	SD	Mean	SD	Mean	SD	Mean	SD	Mean	SD	
Age, years		41.9	11.2	46.7	12.9	46.5	13.1	42.2	13.1	41.9	11.6	<0.001
**Cardiovascular Health metric**												
Smoking, cigarettes/day	1	3.7	9.0	3.7	6.8	3.0	7.3	2.2	6.1	3.4	7.9	0.50
	2	2.6	0.69	2.5	0.42	3.0	0.44	2.5	0.37	3.6	0.65	0.28
BMI, kg/m^2^	1	26.6	5.0	26.5	4.8	26.9	4.9	26.3	5.1	25.7	4.9	0.20
	2	26.7	0.46	26.3	0.28	26.4	0.29	26.8	0.25	26.1	0.43	0.64
Total cholesterol, mg/dl	1	194.3	37.4	203.9	42.2	204.8	43.0	199.6	37.9	204.1	40.6	0.08
	2	196.2	3.9	201.5	2.4	203.9	2.5	202.7	2.1	206.8	3.7	0.05
SBP, mm Hg	1	126.5	17.6	131.6	19.1	130.9	17.1	128.3	17.1	128.1	17.4	0.017
	2	129.7	1.5	130.8	0.89	129.2	0.94	129.4	0.79	129.0	1.4	0.52
DBP, mm Hg	1	80.2	12.0	82.7	10.8	83.4	11.4	82.1	10.6	82.8	11.2	0.11
	2	81.9	1.0	82.3	0.62	82.9	0.65	82.6	0.55	83.2	0.96	0.32
Fasting plasma glucose, mg/dl	1	93.0	22.0	93.7	20.1	93.3	14.8	92.1	16.5	92.4	14.2	0.79
	2	92.6	1.5	92.6	0.93	92.4	0.98	93.4	0.83	93.2	1.5	0.65
Dietary intake score^d^	1	2.3	0.90	2.3	0.96	2.3	0.97	2.2	0.99	2.0	1.0	0.006
	2	2.4	0.09	2.3	0.06	2.3	0.06	2.2	0.05	2.0	0.09	0.001
Physical activity^e^, mins/wk	1	1306	1271	1043	1096	807	884	508	635	296	450	<0.001
	2	1279	88	994	53	827	56	554	47	348	83	<0.001
Total Cardiovascular Health Score, 0–7^f^	1	3.9	1.3	3.6	1.3	3.4	1.4	3.6	1.4	3.5	1.4	0.036
	2	3.8	0.1	3.7	0.0	3.5	0.1	3.5	0.1	3.4	0.1	0.002

*BMI* body mass index, *DBP* diastolic blood pressure, *SBP* systolic blood pressure.

a Includes time spent sitting at place of work, on transportation, at friends', reading, sitting or laying downto watch television or use a computer.

b Model 1: unadjusted means and SD; Model 2: means and SE, adjusted for age, education, gender, income and occupation.

c *P* for linear trend.

d Dietary score of 0–5, based on meeting recommended intakes of fruits and vegetables, fish, fiber-rich whole grains, and minimising intakes of sodium and sugar-sweetened beverages (higher scores indicates better diet).

e Total minutes per week of moderate or vigrorous physical activity.

f Calculated by summing the number of individual metrcis at ideal levels [3].

### Television and Computer Time and Cardiovascular Health

**Figure 2:**
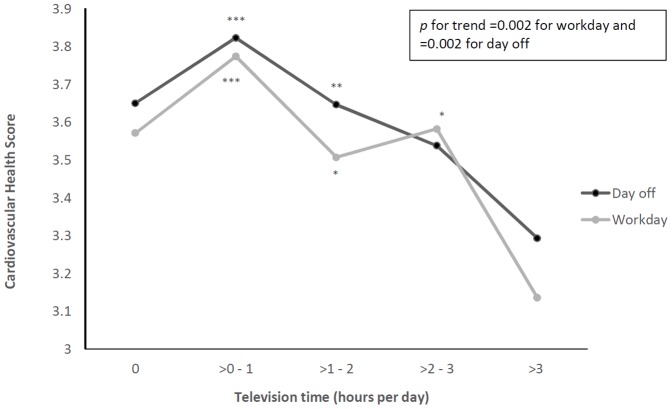
Multivariate adjusted means for Cardiovascular Health Score according to television time on a workday and a day off. Means are adjusted for age, gender, education, income, and occupation. ^*^ *p*<0.05; ^**^ *p*<0.01, ^***^ *p*<0.001 from highest television time group (>3 hours/day).

Figure 2 illustrates the multivariate adjusted means of CHS across increasing categories of television time (including videos/DVD) on both a workday and a day off (ranging from 0 to >3 hours per day). Television time on both days had a significant inverse association with CHS (*p* = 0.002 for both), with adjustment for age, education, gender, occupation and income. The CHS was significantly higher in those who reported less than two hours per day of television time (on both a workday and a day off), compared to those who reported more than three hours of television time. For both days of the week, the CHS was highest in those who watched one hour or less of television, but not none.

**Figure 3:**
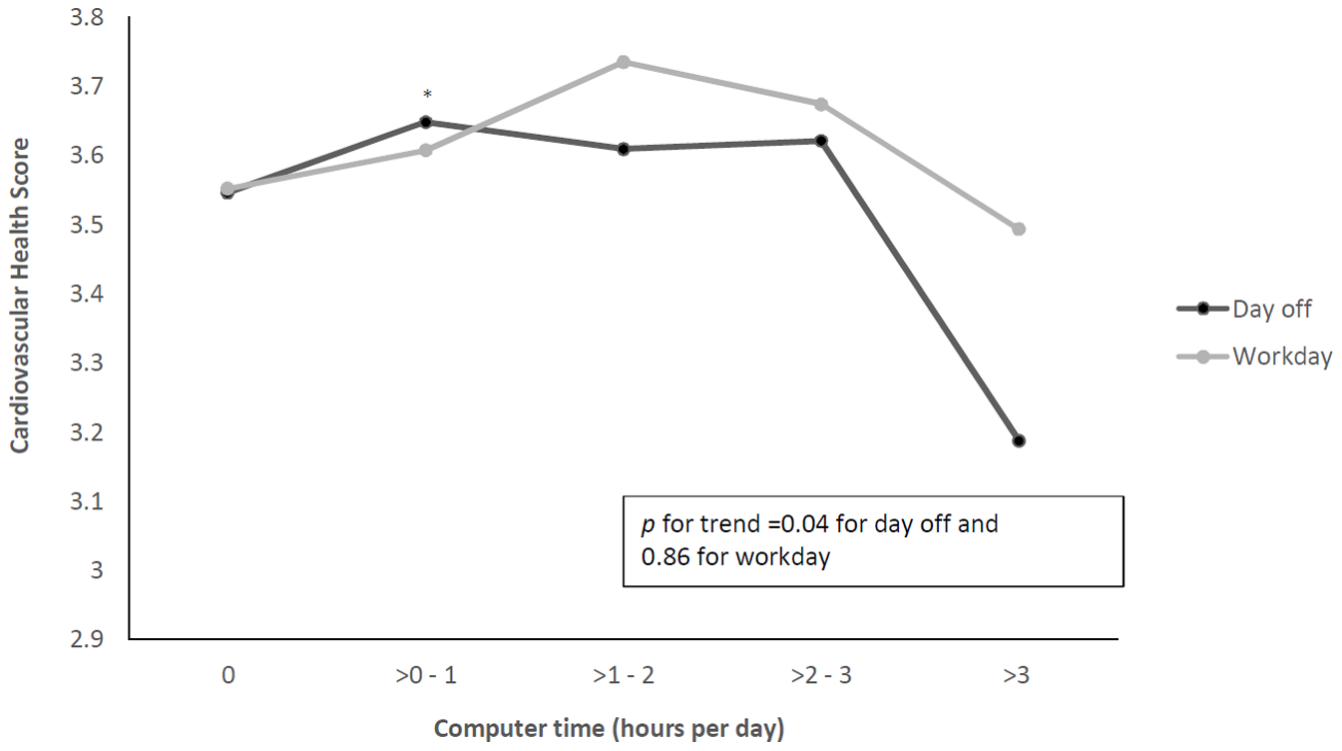
Multivariate adjusted means for Cardiovascular Health Score according to computer time on a workday and a weekend day. Means are adjusted for age, gender, education, income, and occupation. ^*^ *p*<0.05 from highest computer time group (>3 hours/day).

Figure 3 illustrates the multivariate adjusted means of CHS across increasing categories of computer time (including internet and video games) on both a workday and a day off (ranging from 0 to >3 hours per day). A similar inverse relationship was observed between CHS and computer time, only on a day off (*p* = 0.04), with adjustment for age, education, gender, occupation and income. Scores were quite similar for those in front of a computer for between none and three hours, but decreased significantly for those spending more than three hours in front of the screen.

### Sensitivity Analyses

In analyses excluding physical activity from the CHS (modified score ranged from 0 to 6), and including it as a covariate, television time on both a workday and a day off remained inversely associated with the CHS (linear trend *p*<0.001 for both days of week; data not shown). Those in front of the screen for more than three hours per day on either day of the week had the lowest CHS (with adjustment for age, education, gender, income, occupation, and physical activity). The pattern of results between CHS and weekday sitting time and computer time (day off) were similar as to when physical activity was included in the CHS, however these associations were no longer statistically significant.

Linear regression analyses using all sedentary behavior measures as continuous variables (in hours per day), supported the results reported (data not shown). With the same adjustments, the CHS was inversely associated with weekday sitting time (*p*<0.001), television time on a workday and day off (both *p*<0.001), and computer time on a day off (*p* = 0.009).

## Discussion

In this cross-sectional population-based study of European adults, sitting time and time spent viewing television and using a computer, were inversely associated with ideal cardiovascular health, as indexed by seven health factors and behaviors. Higher weekday sitting time (including sitting time in front of the television, at a computer, at place of work, and during transportation) was associated with a lower CHS. When television and computer time were analyzed separately, television time was inversely associated with the CHS, on both a workday and a day off, regardless of age, gender, education, profession type and income. Those watching television for less than two hours per day had significantly better health scores than those watching television for three or more hours per day. For computer time, an inverse relationship was observed on days off, suggesting that participants spending more than three hours per day on a computer during their days off from work are more susceptible to having poor cardiovascular health. From a public health standpoint, this finding is important. Reducing sedentary behavior during ‘free or leisure time’ may be a particularly important message to those who have sedentary or computer-based occupations during the working week.

It should be noted that some of these relationships were not completely linear. For television time on a workday, the CHS was higher for those watching between two and three hours, than for those watching between one and two. Those watching less than one hour per day had higher scores than those watching more than this, but also had higher scores than those watching no television (both workdays and days off). This indicates that perhaps a low level of sedentary time is not necessarily detrimental to health.

The present findings are consistent with other research into sedentary time and clusters of cardiometabolic risk factors. Healy and colleagues [21] found objectively-measured sedentary time was associated with a metabolic risk score, comprised of a cluster of factors (waist circumference, triglycerides, BP, fasting plasma glucose). The study by Ford et al. [29] is one such study that has examined sedentary behavior, including both television and computer time, in relation to metabolic syndrome prevalence among US adults. They found that individuals who did not undertake any moderate to vigorous physical activity during leisure time had almost twice the odds of having the syndrome compared to those engaging in recommended levels (≥150 minutes per week). The likelihood of having metabolic syndrome was highest in those who watched television or videos or used a computer for four or more hours per day outside of work [29]. A recent meta-analysis [28] examined 10 studies which each assessed associations between time spent sedentary and odds of metabolic syndrome, and concluded that higher sedentary time increased the odds of having the syndrome by 73%. Of the 10 studies included in this review [28], only two studies included time sitting other than screen (television or computer) time. It is important to take into account the type of sedentary behaviors that may be associated with cardiovascular health outcomes. Television time and computer time are only two sedentary behaviors that an individual may participate in throughout the day. Using only television time firstly underestimates total sedentary time, and it may not necessarily be an accurate marker of a sedentary lifestyle, particularly in men [42]. It should also not be assumed that television time is spent sitting. Furthermore, a recent large prospective study has provided evidence that different sedentary behaviors may not have the same association with health outcomes [37]. This study found that television time, but not sitting at work or during transportation, was associated with overall and cardiovascular mortality.

It my be postulated that television viewing time may have a greater impact upon health due to coincidental behaviors undertaken at the same time, such as snacking. Studies have shown associations between television viewing time and unhealthy eating, such as higher intakes of high fat and energy dense foods and beverages in both children and adults [43]. Strong positive relationships have been observed for snacking during television viewing and abdominal obesity in women [44]. In the present study, those with the poorest diet scores had the highest weekday sitting times.

This study presents some novel findings and has several strong points. We utilized recent data from a nationwide, population-based sample, with extensive data on cardiovascular risk factors and other potential confounding variables. To the best of our knowledge, this is the first study to have related time spent in sedentary behaviors to the AHA construct of *ideal cardiovascular health*, as opposed to *disease* outcomes, incorporating modifiable lifestyle factors in addition to traditional cardiovascular risk factors. Thus we have extended what is known about sedentary behavior and cardiometabolic health by using a novel outcome measure, the cardiovascular health score, incorporating both health factors and behaviors. Furthermore, we have examined sitting time, television and computer time, and differentiated between the occasion of use (workday versus day off).

Although self-reported measures constitute a limitation, they remain the most feasible and affordable instruments for global surveillance of physical activity. The IPAQ covers the four major domains (work, leissure-time, transport and househould tasks). However, it refers to the seven days preceding the interview, thus may be less accurate in reflecting long-term or seasonal patterns. The cross-sectional design precludes any conclusion regarding causality between sedentary behaviors, and cardiovascular health. Although we controlled for a number of demographic and socioeconomic variables, we cannot rule out the possibility of residual confounding.

Further to previous studies, we have shown that television viewing time regardless of day of the week, and computer time on a day off, were negatively associated with an overall index of cardiovascular health, in this adult European sample of men and women. This suggests that sedentary behaviors during leisure time away from occupational sitting time, may be a relevant indicator of cardiovascular risk. We opted to include physical activity in the CHS, as defined by the AHA. There is evidence to show that any negative impact upon health from time spent sedentary is unlikely to be due to confounding from time spent undertaking physical activity [37]. A number of studies have shown independent associations between sitting time and total mortality, regardless of physical activity level [45], and that individuals can participate in high intensity physical activity, while spending the majority of the day undertaking sedentary behaviors, and still meet physical activity recommendations [18], [46]. The present study has also demonstrated independent associations between television time and cardiovascular health, as inverse associations between the two remained when physical activity was removed from the CHS and controlled for.

In the same light, future research in this area should ensure that the terms sedentary and inactive are clearly defined and distinguished [17]. It should be noted that this study sample could be considered active, with 70.4% meeting the AHA recommendations of at least 150 minutes per week of moderate intensity physical activity [3] (mean of 766 [±935] minutes per week).

Future studies should explore whether different sedentary behaviors have differential associations with health outcomes, particularly utilizing both self report and objective measures of sedentary time and energy expenditure [47]. It will be important to explore new means and effective measures to reduce sitting time during the working week [48], [49]. Interventions implemented in the workplace have been successful in reducing sitting time in the short-term [50], but larger, longer-term studies are needed. Our findings also indicate that targeting a reduction in sedentary behaviors on days off, such as time spent watching television and on a computer, may be just as important as interventions aimed at reducing sitting time during the week. As recognized by others [43] it would also appear important to further examine longitudinal associations between different types of sedentary behavior, such as time spent at a computer, with dietary patterns, and whether interventions aimed at reducing time undertaking sedentary behaviors also influence poor dietary habits.

### Conclusions

The findings from this cross-sectional, explorative study indicate that time spent in sedentary behaviors during both workdays and days off, may be associated with poorer levels of *ideal* cardiovascular health. The findings are consistent with the growing literature suggesting that reducing overall sedentary time may be important for the prevention of cardiovascular health problems. As mortality and morbidity from CVD continue to have a major social and economic impact in Europe, interventions aimed at risk factor reduction will be important. The modification of unhealthy lifestyle behaviors (physical inactivity, smoking, dietary intakes high in saturated fats and added sugars), as recommended by the AHA in order to improve cardiovascular health in the population as a whole, will continue to be important to complement traditional medical approaches to CVD management [3]. Large randomized trials incorporating physical activity interventions and techniques to reduce screen time will be important for future research in this area.
